# Character Growth Following Collective Life Events: A Study on Perceived and Measured Changes in Character Strengths During the First Wave of the COVID-19 Pandemic

**DOI:** 10.1177/08902070211040975

**Published:** 2021-08-27

**Authors:** Fabian Gander, Lisa Wagner

**Affiliations:** 1 Department of Psychology, University of Basel, Basel, Switzerland; 2 Department of Psychology, 27217 University of Zurich, University of Zurich, Zurich, Switzerland

**Keywords:** Character strengths, posttraumatic growth, COVID-19, personality change, compliance

## Abstract

Did the COVID-19 pandemic promote character growth? Studies using sequential samples suggest that collective life events can result in character growth, but their conclusions have been questioned. This study used three approaches to examine character growth during the first wave of the pandemic: perceived changes in oneself and in a close other, and a longitudinal analysis of changes. In addition, we tested whether character strengths assessed before the pandemic predicted specific instances of growth, that is, engagement in volunteering and compliance with regulations during the pandemic. German-speaking participants (*N* = 366, 76.5% female, mean age: 45.33 years) who had completed an assessment of character strengths before the pandemic reported on perceived changes in character strengths, engagement in volunteering, and compliance with regulations. A subsample also completed a second assessment of character strengths. The results showed that (a) participants reported perceived changes for most character strengths in both themselves and close others, while (b) longitudinal increases were only observed for humility and prudence. Pre-pandemic character strengths predicted (c) engagement in volunteering and (d) compliance with regulations. We conclude that actual character growth was smaller than the perceived changes but that the character strengths did predict relevant behaviors related to the COVID-19 pandemic.

Character strengths comprise a set of 24 positively valued personality traits (Peterson & Seligman, 2004). Compared to broader, more abstract concepts such as the Big Five, character strengths are more specific traits that are situated on a hierarchically lower level of personality (e.g., Ashton et al., 2014). Another main difference from traditional conceptualizations of personality is the explicit expectation that character strengths are malleable, by such factors as deliberate training and learning experiences, or by important life events (Peterson & Seligman, 2004). The literature has long suggested that such changes in character strengths (or character growth) may take place following major adverse historical events (Peterson & Seligman, 2003), recovery from illness (Peterson et al., 2006), or traumatic experiences (Peterson et al., 2008; Schueller et al., 2015). These studies have advanced the idea that adverse life events might, despite their apparent undesirable effects, also contribute to growth, and that this growth may be reflected in changes in character strengths. All of these studies, however, suffered from obvious methodological limitations. Since most life events, in particular adverse ones, are not highly predictable (e.g., Kritzler et al., 2021), it is difficult to conduct studies that compare individuals’ traits of interest before and after an event, especially if a study is to use sufficiently large samples. Thus, the previous studies either only relied on post-event assessments of character strengths or subjectively perceived changes in character strengths, or they used sequential sample designs (i.e., non-overlapping samples for pre- and post-event assessments).

The first wave of coronavirus disease 2019 (COVID-19) and the associated restrictions set out by most governments worldwide led to major changes in most people’s everyday lives (e.g., Mækelæ et al., 2020). The COVID-19 pandemic, therefore, could be considered a major life event that might elicit character growth. As with the events examined in earlier studies (e.g., traumatic events), the pandemic was also unforeseen, but this event differed in that it affected most people simultaneously. This created circumstances that are ideal for conducting a longitudinal study using the same set of participants. In the present study, we focused specifically on the period of time at the beginning of the first wave of the COVID-19 pandemic during Spring 2020 in which major restrictions on everyday life were in place. In the German-speaking countries, where we collected data, these restrictions began in mid-March and were gradually relaxed by the end of April/the beginning of May (Hale et al., 2021). Henceforth, we will be referring to this time period as the (first wave of the) “COVID crisis.”

The main goal of the current study was to investigate character growth in the context of the COVID-19 pandemic to identify any changes in character strengths and to determine whether character strengths assessed before the crisis predicted compliance with regulations and engagement in volunteering during the crisis. Furthermore, we examined whether the extent to which an individual was affected by the crisis was related to changes in character strengths. Finally, we were interested in whether longitudinal findings on changes in character strengths were comparable to approaches that only examined post-event assessments or subjectively perceived changes in character strengths.

## Stability and Changes in Character Strengths

Peterson and Seligman (2004) suggested that character strengths are both stable and malleable. While some studies have shown that character strengths are relatively stable in their rank-order and mean-level stability over longer periods of time (Chopik et al., 2021; Gander et al., 2020), other studies have suggested normative development of character strengths. For example, Martínez-Martí and Ruch (2014) used cross-sectional data to uncover small relationships with age for some character strengths and suggested that these relationships might indicate the contribution of character strengths in adaptation to different life stages. Similarly, Baumann et al. (2020) reported differences between particular character strengths among employed and retired persons. They suggested that mean-level differences in character strengths might be reflective of normative age-related changes. Finally, while a few studies have used longitudinal data to study changes in character strengths (e.g., Chopik et al., 2021), little is known regarding longitudinal changes in character following major life events.

## Changes in Character Strengths in Response to Life Events

Based on a review of the previous literature, Tedeschi and Calhoun (1996) described five dimensions of benefits people might experience following traumatic events, which included increases in relating to others, seeing new possibilities, growth in personal strength, spiritual changes, and increased appreciation of life. These dimensions show a striking resemblance to several character strengths (e.g., relating to others: kindness, love, teamwork; new possibilities: curiosity, hope, love of learning, zest). Thus, it seems clear that the changes that may follow trauma that have been described in the literature are related to character. We are not arguing, however, that these are interchangeable concepts; the dimensions of posttraumatic growth are not trait-like, stable patterns of behavior. Instead, these dimensions describe insights or cognitions that are gained through specific experiences that might be considered mechanisms that promote character growth. For example, mastering a crisis might lead to the discovery that one is stronger than one had thought (personal strength), which might then lead to an increase in the character strength of bravery. Furthermore, several studies have addressed the relationship between critical life events, character strengths, and posttraumatic growth. One early study conducted by Peterson and Seligman (2003) addressed whether changes in character might occur following life events. This study used a cross-sectional sequential sample design to compare character strength self-assessments at several points in time before and after the terrorist attacks that happened in the United States on September 11, 2001. The authors found higher scores in seven character strengths (i.e., gratitude, hope, kindness, leadership, love, spirituality, and teamwork) in the samples assessed following the terror attacks, which led them to conclude that character strengths might indeed be malleable. Three later studies corroborated these findings. The first of these used a retrospective cross-sectional design to compare the character strength scores of those who had recovered (either fully or partly) from physical illnesses or psychological disorders (Peterson et al., 2006) with the scores of those who had not recovered or had not been affected by such diseases or disorders. The results of this study showed group differences for several character strengths, including appreciation of beauty and excellence, bravery, creativity, curiosity, fairness, forgiveness, gratitude, humor, kindness, love of learning, and spirituality. The authors concluded that “illness and disorder take a toll on both strengths of character and life satisfaction” but that “recovered individuals may then show elevated strengths of character” (Peterson et al., 2006, p. 25). The second study (Peterson et al., 2008) used a retrospective cross-sectional design to examine the associations between character strengths with the number of traumatic events (e.g., life-threatening accidents or illness, physical attacks) a person reported having experienced. The authors report positive relationships between the number of traumatic experiences and the strengths of creativity, curiosity, love of learning, bravery, perseverance, honesty, zest, kindness, leadership, appreciation of beauty and excellence, and spirituality. Peterson et al. (2008) concluded that character growth might occur following the experience of trauma. The third study considered the possibility that character strengths might not only be affected by the experience of adverse events, but by positive events experienced collectively. Proyer et al. (2014) used a sequential sample design to study group differences in character strengths at several points in time before and after the 2008 European Football Championship in Switzerland (one of the hosting countries). The participants assessed following the championship reported higher scores in honesty, fairness, humility, and spirituality than those assessed before the event.

While the findings of these studies are promising and advocate for the malleability of character following life events, they are somewhat tempered by the findings of a later study by Schueller et al. (2015). This study also used a sequential sample design to study people living close to tragic events (i.e., school shootings), in this case, through a direct comparison of three different school shootings. The results across the events were widely inconsistent, which led Schueller et al. (2015) to point out the possibility that the conclusions of previous studies might have been premature, and that character strengths may not change in a systematic way following experiences of adversity. Along the same lines, Lamade et al. (2020) examined self-reports of character strengths before, between, and after terror attacks in France (sequential samples) and compared the results with data collected in the United States and Australia during the same time periods. The authors reported “no discernable pattern of results” and concluded that “the use of sequential samples as a proxy for longitudinal prospective samples … should be undertaken and interpreted with great caution” (Lamade et al., 2020, p. 298).

Jayawickreme et al. (2021) summarized that there is little empirical evidence so far that mean-level changes in character strengths occur following adversity, especially given the reliance on cross-sectional data and the lack of prospective longitudinal research on character strengths. These issues are not specific to research on character strengths; they also involve research on changes following trauma in general. As Jayawickreme and Zachry (2018) summarized in a recent review, “the extant literature provides a number of theoretical perspectives but little evidence for positive personality change following adversity, given the methodological limitations in much of the research” (p. 458). They went on to mention the need for more research using longitudinal data.

Further to this, it has been shown that self-perceived changes often only have a small relationship with actual change (Jayawickreme & Blackie, 2014). For example, Frazier et al. (2009) examined the relationship between perceived growth and actual growth in the dimensions described by Tedeschi and Calhoun (1996) by comparing pre- and post-event assessments. Their results suggested that perceived growth only showed small relationships to actual growth. Frazier et al. (2009) concluded that retrospective assessments do not measure actual growth and asserted that actual growth and perceived growth represent different processes. Many previous studies were further limited by their sole reliance on self-reports of growth. Jayawickreme and Blackie (2014) argued that close other persons might be better informants about posttraumatic growth, as they should be able to observe changes while being less prone to bias in their responses. In the present study, we explored this idea by including reports on close others as well as self-reports of posttraumatic growth.

## Changes in Prosocial Behavior Following Life Events

Growth following trauma might also entail changes in specific behaviors. Vollhardt (2009) argued that adverse life events may enhance the motivation to support other people, be it on an interpersonal (i.e., helping a specific other person) or on a collective level (i.e., helping a group or the society). This concept of “altruism born of suffering” (Vollhardt, 2009, p. 53) closely relates to Tedeschi and Calhoun’s (1996) notion of posttraumatic growth which covers having compassion for others and investing in relationships as part of the domain of “relating to others”. Several studies provided empirical support for this notion. For instance, Frazier et al. (2013) showed that individuals who experienced a recent trauma reported engaging more often in daily helping behavior than those who did not experience a trauma.

The idea that adverse events might trigger specific prosocial behaviors could also be relevant in the context of the COVID crisis: One of the challenges during the crisis was providing the necessary support to community members, especially those who were at particularly high risk for severe forms of COVID-19 and, therefore, were explicitly discouraged from leaving their homes. In many cases, neighbors directly offered help to those in need of support (e.g., by buying and delivering groceries), while some communities also organized networks for volunteers to offer their help, which helped to close critical gaps in public services (Churchill, 2020; Miao et al., 2021). In Vollhardt’s (2009) terminology, such voluntary engagement can be considered an instance of interpersonal helping.

Further, one might argue that compliance with regulations (e.g., mask wearing, limiting personal contacts) can be considered an instance of collective helping as behaving according to the regulations reduces the risk of infection for others. Pfattheicher et al. (2020) demonstrated that compliance related to and was fostered by empathy, in line with Tedeschi’s (1999) suggestion that experiencing adversity and “recognizing one’s vulnerability, may be a kind of empathy training” (p. 323) which, in turn, may promote helping. Thus, we consider both volunteering and complying with regulations as specific instances of growth in the context of the COVID crisis.

## Character Strengths and Specific Instances of Growth (Voluntary Engagement and Compliance)

For the present study, we were interested in whether individual differences, such as character strengths, predict such specific instances of growth during the crisis. Knowledge about who will show voluntary engagement and comply (or fail to comply) with official regulations might be highly relevant for tailoring health-related campaigns to specific groups.

Regarding voluntary engagement, Zettler et al. (2021) found that feelings of social cohesion, which might be understood as a precursor for volunteering, were associated with honesty-humility, extraversion, and agreeableness. We could not find any research that studied individual difference variables as predictors of volunteering related to the COVID-19 pandemic. We argue, however, that it should be added to the range of pandemic-related behaviors of interest because of its relevance for communities and individuals.

Given their positive valence and moral value, we assume that character strengths should be particularly suited to predict who will engage in volunteering. Those high in the character strengths assigned to the virtue of humanity (i.e., love, kindness, and social intelligence) and teamwork should have a stronger inclination to contribute to their community by helping others. In fact, in their descriptions of character strengths, Peterson and Seligman (2004) explicitly mention volunteering in relation to kindness, teamwork, and spirituality. Moreover, all character strengths assigned to the virtue of humanity are related to prosocial behavior. Furthermore, we assume that the character strengths of bravery, zest, and hope would be conducive to initiating volunteering during a pandemic, as it requires initiative and an optimistic outlook; these strengths are described as being of relevance when individuals are faced with difficult circumstances (Peterson & Seligman, 2004).

Regarding compliance, several studies have looked at the associations between personality traits and specific compliance behaviors during the COVID crisis. For example, Aschwanden et al. (2021) examined the associations of the Big Five dimensions with precautions (e.g., using hand sanitizer) and preparations (e.g., buying face masks). Their results showed that taking precautions was positively related to higher conscientiousness, extraversion, openness, and agreeableness and negatively related to neuroticism, while making preparations was related positively to extraversion. Zajenkowski et al. (2020) examined compliance (i.e., the extent to which one complied with governmental regulations) in association with the Big Five, the dark triad, and situational characteristics. They found that compliance went along with agreeableness, but also found that situational characteristics were better predictors of compliance than personality traits. Zettler et al. (2021) found honesty-humility to be most strongly related to behavioral adjustment (i.e., following recommendations, practicing hygiene, and physical distancing) across the HEXACO personality traits.

We argue that character strengths are especially well-suited to predict compliance since they focus on desired, moral, positively valued behavior (Stahlmann & Ruch, 2020) and are more specific than broad personality traits. Strengths such as prudence (i.e., being careful about one’s choices and not taking unnecessary risks), humility (i.e., not seeking the spotlight, not regarding oneself as more special than others), and teamwork (i.e., being a loyal member of a group) might be especially relevant for following official guidelines. This idea was confirmed by Proyer et al. (2013), who examined the cross-sectional relationships of character strengths with health-related compliance in general (e.g., complying with medical prescriptions or doing regular health checks). Their findings showed positive associations with several character strengths (i.e., honesty, love, kindness, social intelligence, teamwork, and prudence). We expect that these cross-sectional findings can extend to the prediction of future compliant behavior.

## The Present Study

In the present study, we aimed to examine character strengths growth, volunteering, and compliance during the COVID crisis in German-speaking areas. We used three different operationalizations of changes in character strengths (i.e., self-perceived changes, perceived changes in close others, and measured differences in character strengths before and after the first wave of the crisis) to overcome the shortcomings of previous approaches and to allow comparison between the convergence of the three approaches. We asked participants (1) whether they (retrospectively) think that their character strengths had changed. In order to reduce desirability biases (in line with Jayawickreme and Blackie’s (2014) suggestion), we also asked participants (2) to consider retrospectively whether the character strengths of a close other person had changed. If the COVID crisis had a general effect on specific character strengths, this should have also been observed in close others. Usually, researchers ask close acquaintances of the participants to provide information about the participant in order to avoid desirability biases (e.g., Blackie et al., 2015). For this study, we intentionally reversed this logic and asked participants to provide information about close acquaintances. If the COVID crisis as a collective life event indeed evoked changes in specific character strengths, we would expect that these changes could also be measured by asking participants to provide information on a close other who they have been in close contact with during the crisis, while these ratings should, at the same time, be less prone to desirability biases. This was an exploratory research question and we wanted to investigate whether this approach would yield results that are more similar to the results of actually measured differences and could be used as an alternative to conventional informant reports. Finally, we (3) analyzed self-assessments of character strengths that had been completed up to 1.5 years before the COVID crisis; we invited the same set of participants to complete the same instrument again after the first wave of the COVID crisis and computed the differences between the two measurement periods.

We identified five research questions for the current research. While Research Questions 1 to 2 were preregistered (see procedure), Research Questions 3 to 5 were formulated after the preregistration. Research Question 1 was aimed at examining perceived changes in character strengths following the first wave of the COVID crisis. We analyzed whether the participants had perceived changes in character strengths in oneself and in a close other and whether they reported differences between character strengths before and after the first wave of the crisis. We did not formulate specific hypotheses, but we expected that changes in several strengths would be observed, in line with earlier research (e.g., Peterson et al., 2008).

Research Question 2 examined whether the degree to which the COVID crisis impacted participants was related to perceived changes (in oneself and others) and measured changes in character strengths. We examined this research question on an exploratory basis and formulated no specific hypotheses.

Research Question 3 focused on comparing our results with those of earlier studies on character strengths and posttraumatic growth (in particular, the work of Peterson et al., 2008). Thus, we were interested in whether changes in character strengths before and after an event show the same relationships to posttraumatic growth as when character strengths are only examined after an event (as proposed by Peterson et al., 2008). This comparison allowed us to determine whether longitudinal data is required to address research questions on the impact of specific events or whether post-event assessments or asking people retrospectively about perceived changes can be considered adequate proxies for longitudinal data.

Research Question 4 examined whether character strengths can predict compliance. We studied the relationships between character strengths reported before the crisis and compliance with official governmental measures and recommendations. In line with theoretical considerations and earlier findings (Proyer et al., 2013), we expected to find positive relationships for both teamwork and prudence.

Research Question 5 investigated whether character strengths can predict engagement in volunteering. Specifically, we studied whether character strengths assessed before the crisis predicted whether individuals engaged in volunteering during the COVID crisis. Building on theoretical considerations (Peterson & Seligman, 2004), we expected to find that bravery, zest, love, kindness, social intelligence, teamwork, hope, and spirituality were higher in individuals who engaged in volunteering.

## Method

### Participants

Power analysis suggested that a sample size of at least *N* = 160 participants was necessary to observe a small to medium effect (*r* = .20, power = .80, one-tailed α = .05). We contacted participants who had completed the VIA Inventory of Strengths (Peterson et al., 2005) on a public website up to 1.5 years before the COVID crisis began (i.e., between July 2018 and December 2019) and invited them to participate in this study. We collected data between the end of June 2020 and the end of August 2020, and the sample size was determined by the number of participants during this time. A total of 372 participants completed the survey, five of whom were excluded because they completed the survey unusually fast (using the suggested cut-off by Leiner, 2019). Another person was excluded because they failed to provide information on age and gender, both of which were subsequently used as control variables. The final sample consisted of *N*_self_ = 366 participants (76.5% women) aged 20 to 82 (*M* = 45.37, *SD* = 12.62). Participants were predominantly living in Germany (56.8%), Switzerland (31.7%), or Austria (9.3%), and all had a good command of the German language. Most participants (84.7%) were currently working. The sample was well-educated, with 69.1% holding a degree from a university or a university of applied sciences. Most of the participants (76.5%) lived with other people (i.e., friends, partner, relatives, or children), while 23.0% lived alone.

Most of the participants (*N*_peer_ = 337) also reported on perceived changes in a close other (i.e., a person they lived with or, if they were living alone, the person with whom they had the most frequent and closest contact during the crisis). The sample of close others (46.6% women) ranged between 7 and 96 years in age (*M* = 45.24, *SD* = 17.51), and the participants had known the other person for an average of *M* = 20.67 years (*SD* = 14.8). Most of the participants (82.6%) had interacted with the other person several times per day during the crisis, and all of them had interacted with the other person at least once per week (the data for the close others was removed if they had interacted less frequently than once per week).

A self-selected subsample (*N*_VIA changes_ = 150) completed the VIA-IS again, after the first wave of the crisis. This subsample did not differ from the full sample with regard to age (*t*[329.91] = 0.93, *p* = .354), gender (*χ*^2^[1, *N* = 150] = 3.77, *p* = .052), education (*χ*^2^[6, *N* = 150] = 4.2, *p* = .650), or living arrangement (*χ*^2^[1, *N* = 150] = 0.6, *p* = .437).

### Instruments

The Character Strengths Change Rating Form – Self (CSCRF-S) assesses perceived changes in the 24 character strengths described in the VIA classification as a consequence of the first wave of the COVID crisis with one item per character strength. The CSRF-S was developed based on Ruch et al.’s (2014) Character Strengths Rating Form. A sample item for the character strength of creativity is “creativity describes the pronounced tendency, for example, to think about new ways of solving problems and to have creative and original ideas without being satisfied with conventional solutions when better possibilities are available.” Each item was introduced with “compared to the time before the COVID crisis, I am now …” and the participants were asked to record their responses on a 7-point Likert-style scale that ranged from 1 (*much less than before*) to 7 (*much more than before*), where 4 indicated *no change*. The participants completed this instrument both for themselves and for a close other person (Character Strengths Change Rating Form – Peer; CSCRF-P).

The VIA-IS (Peterson et al., 2005; German version by Ruch et al., 2010) assesses the 24 character strengths from the VIA classification as traits with 10 items per strength. It uses a 5-point Likert-style scale that ranges from 5 (*very much like me*) to 1 (*very much unlike me*). A sample item for curiosity is “I find the world a very interesting place.” All participants had completed the measure before the COVID crisis, and a subset of the participants completed it again after some of the restrictions during the first wave of the pandemic had been eased. Internal consistencies in the present study ranged from α = .70/.70 to α = .91/.93 (median α = .78/.80) for the first and second assessment, respectively.

The Posttraumatic Growth Inventory (PTGI; Tedeschi & Calhoun, 1996; German version by Maercker & Langner, 2001) assesses five aspects of posttraumatic growth (i.e., relating to others, new possibilities, personal strength, spiritual change, and appreciation of life). For the present study, we adapted the instruction for the scale to specifically address changes following the first wave of the COVID crisis. The scale consists of 21 items rated on a 6-point Likert-style scale ranging from 1 (*I did not experience this change as a result of the COVID crisis*) to 6 (*I experienced this change to a very great degree a result of the COVID crisis*). A sample item is “I can better appreciate each day.” Since preliminary analyses suggested that the original subscales were highly correlated, we only reported the total score across all items. Internal consistency was α = .95. While the participants completed eight additional items for changes specific to the COVID crisis, we excluded these items due to their high empirical overlap with existing items and only report the results of the original scale.

The individual impact of the COVID crisis was assessed as an index of crisis-related changes that participants might have experienced. This index covers 20 aspects and allows for a more objective assessment of the crisis’s impact on the participants’ everyday lives. Item examples are “I experienced changes in my income” and “I experienced changes in the frequency of social contacts.” All of the items were answered on a 2-point scale (0 = *No*; 1 = *Yes*). The items are available at an online repository (https://osf.io/atc48/). All of the answers were summed to give a total index score.

We also assessed participants’ compliance with government regulations. Because the regulations had strong differences in intensity and duration depending on where the participants lived, we asked them to report on the extent to which they followed the regulations in general rather than explicitly specifying them. This item (“to what extent have you complied with the official measures and recommendations of the government?”) was rated on a 6-point Likert-style scale that ranged from 1 (*never*) to 6 (*always*).

Finally, we asked the participants about their engagement in volunteering during the crisis. We asked the participants “have you volunteered in any form? (e.g., shopping for neighbors)” and they had the option of selecting “no” or “yes.” If they replied yes, they were asked to specify the type of voluntary engagement. A total of 37.7% of the participants reported that they had volunteered during the crisis. Most of the volunteer activities reported involved helping within the neighborhood (mainly with shopping and childcare), but they also included more extensive forms of volunteering, such as offering telephone counseling.

### Procedure

The study and its main research questions (1 and 2) were preregistered at https://aspredicted.org/mw3aq.pdf. According to the local university guidelines, the present study was exempt from ethical review and did not require formal approval. We invited participants to complete an online survey that covered subjective changes in character strengths (CSCRF-S), individual impact of the crisis, compliance with government regulations, engagement in volunteering during the crisis, and additional measures not presented here. Then they were asked to complete a questionnaire (CSCRF-P) about a person they live with or with whom they had the most frequent and the closest contact during the crisis. Finally, they were invited to complete the VIA-IS again. Participation was voluntary and participants provided written consent. They were given the option to receive feedback on the study results and, if they completed the VIA-IS again, individual feedback on changes in their character strengths. The average time lag between completing the VIA-IS for the first time and participating in this study was *M* = 595 days (*SD* = 83; range = 225–738 days). Further to this, a small amount (1 CHF) was donated to the Swiss Red Cross on behalf of each participant. All data, syntax, descriptive statistics, and zero-order correlations among the study variables presented in this article are available online at https://osf.io/atc48/.

### Data Analysis

For the analyses regarding perceived changes in oneself, we controlled for gender and age. For the analyses regarding perceived changes in close others, we controlled for the gender and age of the close other. As in previous research on this topic, we used difference scores (i.e., we subtracted the VIA-IS raw scores at time one from those at time two) for our analysis of the changes in the assessments of character strengths before and after the first wave of the COVID crisis (Frazier et al., 2009). We controlled for gender, age, and duration since the first assessment for the analyses involving difference scores. All covariates were z-standardized. Statistical significance for effect sizes (Cohen’s *d* or correlations) were determined by *p*-values; additionally, confidence intervals are given in the figures.

## Results

### Research Question 1: Changes in Character Strengths

**Figure 1.**
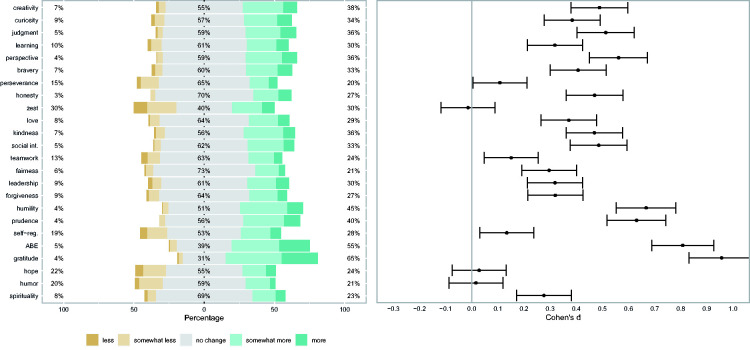
Perceived Changes in Oneself in Character Strengths Following the COVID Crisis. *Note. N* = 365. The panel’s left side shows response frequencies (“less”/“much less” and “more”/“much more” were collapsed). The panel’s right side shows the effect sizes (based on regression weights and controlled for gender and age) of the mean scores with associated 95% confidence intervals. Learning = love of learning. Social int. = social intelligence. Self-reg. = self-regulation. ABE = appreciation of beauty and excellence.

**Figure 2. fig2-08902070211040975:**
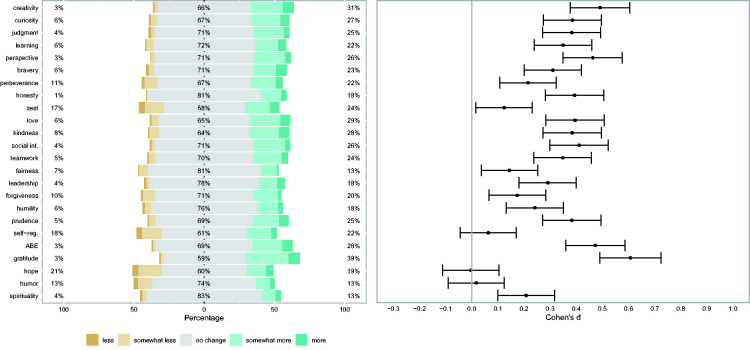
Perceived Changes in Close Others in Character Strengths Following the COVID Crisis. *Note*. *N* = 339–340. The panel’s left side shows response frequencies (“less”/“much less” and “more”/“much more” were collapsed). The panel’s right side shows the effect sizes of the mean scores (based on regression weights and controlled for gender and age) with associated 95% confidence intervals. Learning = love of learning. Social int. = social intelligence. Self-reg. = self-regulation. ABE = appreciation of beauty and excellence.

Results for perceived changes in oneself and others, and differences between character strengths before and after the first wave of the crisis are given in Table 1, and in Figures 1 [Fig fig2-08902070211040975]to 3.

**Table 1.** Research Question 1: Changes Perceived in Oneself and Others, and Differences in Character Strengths (Figures 1–3).

	Perceived changes in oneself		Perceived changes in close others		Differences in character strengths	
	Cohen’s *d*	*t*(362)	Cohen’s *d*	*t*(333)	Cohen’s *dz*	*t*(146)
creativity	0.49	9.30*	0.49	8.97*	–0.03	–0.40
curiosity	0.38	7.31*	0.39	7.04*	–0.05	–0.63
judgment	0.51	9.76*	0.38	6.98*	0.02	0.30
learning	0.32	6.06*	0.35	6.38*	–0.03	–0.42
perspective	0.56	10.69*	0.46	8.45*	0.07	0.89
bravery	0.41	7.74*	0.31	5.66*	0.09	1.11
perseverance	0.11	2.05*	0.22	3.93*	0.02	0.29
honesty	0.47	8.95*	0.39	7.18*	0.06	0.67
zest	–0.01	–0.27	0.12	2.26*	–0.01	–0.10
love	0.37	7.07*	0.40	7.21*	–0.07	–0.90
kindness	0.47	8.94*	0.38	7.02*	–0.16	–1.92
social int.	0.49	9.25*	0.41	7.51*	–0.02	–0.25
teamwork	0.15	2.87*	0.35	6.36*	–0.07	–0.84
fairness	0.30	5.64*	0.14	2.64*	–0.03	–0.36
leadership	0.32	6.07*	0.29	5.31*	–0.06	–0.77
forgiveness	0.32	6.08*	0.17	3.19*	–0.06	–0.73
humility	0.67	12.70*	0.24	4.41*	0.17	2.11*
prudence	0.63	12.00*	0.38	6.99*	0.33	3.99*
self-reg.	0.13	2.55*	0.06	1.16	0.15	1.79
ABE	0.81	15.36*	0.47	8.63*	–0.06	–0.73
gratitude	0.96	18.20*	0.61	11.08*	0.00	–0.06
hope	0.03	0.54	0.00	–0.06	0.12	1.47
humor	0.02	0.30	0.02	0.32	0.00	–0.01
spirituality	0.28	5.26*	0.21	3.81*	–0.16	–1.88

*Note. N* = 365/339/150. Learning = love of learning. Social int. = social intelligence. Self-reg. = self-regulation. ABE = appreciation of beauty and excellence. Analyses are controlled for gender and age (Columns 1–4), and additionally duration since the first character strengths assessment (Columns 4–6).

*p < .05.

For perceived changes in oneself and others, we computed a series of linear regressions to analyze whether the perceived changes in oneself and others differed from the scale midpoint (=4, indicating no change). Since we were interested in whether the perceived changes differed from the scale midpoint of 4 (=“no change”), we subtracted 4 from the perceived change scores. To account for the effects of age and gender, we then predicted these scores by (z-standardized) gender and age in a linear regression. We then checked whether the intercept of these regressions differed from 0. We used the (z-standardized) covariates to predict the perceived change and tested whether the intercept differed from the scale midpoint for each character strength. The results for perceived changes in oneself (CSCRF-S) are shown in Figure 1. Figure 1 demonstrates that participants perceived increases (in themselves) for all of the character strengths except zest, hope, and humor, for which no changes were reported. They reported the most pronounced changes for gratitude, appreciation of beauty and excellence, humility, and prudence. The results for perceived changes in close others (CSCRF-P) are shown in Figure 2. The participants perceived increases for most of the character strengths in their close others, with the exception of humor, hope, and self-regulation. The most pronounced changes were perceived in gratitude and appreciation of beauty and excellence.

**Figure 3.**
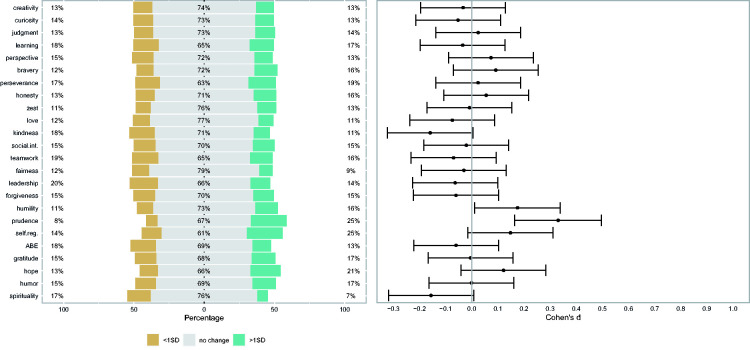
Changes in Character Strengths Following the COVID Crisis. *Note. N* = 150. The panel’s left side shows response frequencies (“less”/“much less” and “more”/“much more” were collapsed). The panel’s right side shows the effect sizes of the mean scores (based on regression weights and controlled for gender, age, and duration since the first character strengths assessment) with associated 95% confidence intervals. Learning = love of learning. Social int. = social intelligence. Self-reg. = self-regulation. ABE = appreciation of beauty and excellence.

To examine whether there were differences in character strengths before and after the first wave of the crisis, we predicted the difference score for each character strength by the covariates and tested whether the intercept differed from zero in a series of regression analyses. As shown in Figure 3, the analyses did not show any differences between the VIA-IS before and after the first wave of the crisis for most of the character strengths; only the prudence and humility scores showed an increase. A direct comparison of the self-perceived and the measured differences between the two assessments showed that they were unrelated (the median of the associations across the corresponding strengths was *r*[148] = .07).

Further, when computing rank-order correlations of the sample-average estimates of change across all three data sources (i.e., correlations of the three columns with Cohen’s d measures in Table 1) we found a high convergence between the changes perceived in oneself and in others (*r_s_*[22] = .81), but both were unrelated to the measured differences (*r_s_*[22] = .15, and *r_s_*[22] = –.16 for perceived changes in oneself and others, respectively).

### Research Question 2: Effects of Individual Impact of the Crisis on Character Strengths

For this research question, we ran the same analyses we conducted for Research Question 1. For this question, however, we added the individual impact of the crisis as an additional predictor (or moderator for differences between before and after the first wave of the crisis). The participants reported having experienced between one and 17 of the 20 crisis-related changes (*M* = 8.36; *SD* = 2.93). We then summed the responses to form an index of the individual impact of the crisis. Inconsistent patterns of results emerged, and because of this, we only give a brief summary of the findings in the following.

We examined whether the individual impact of the crisis was related to perceived changes in oneself (CSCRF-S) and to differences in character strengths before and after the first wave of the crisis. The results (see Table 2 and Figure A in the online repository) revealed small effects for perceived changes in oneself in honesty and no relationship with measured differences in strengths. The results for measured differences in strengths also remained unchanged when we only analyzed the reports of the participants who were more strongly affected by the crisis (i.e., those who scored above the median in the impact index).

**Table 2.** Research Question 2: Relationships Between Changes in Character Strengths and Impact of the Crisis (Figure A in Online Repository).

	Perceived changesin oneself		Differences in character strengths	
	Cohen’s *d*	*t*(362)	Cohen’s *dz*	*t*(145)
creativity	0.10	1.92	–0.11	–1.30
curiosity	0.06	1.08	–0.06	–0.78
judgment	–0.03	–0.48	0.08	1.00
learning	0.04	0.70	–0.02	–0.18
perspective	0.04	0.79	0.02	0.25
bravery	0.03	0.50	–0.02	–0.18
perseverance	–0.07	–1.27	–0.05	–0.56
honesty	0.13	2.47*	0.07	0.82
zest	–0.04	–0.78	–0.07	–0.89
love	0.02	0.37	–0.14	–1.65
kindness	–0.03	–0.62	–0.07	–0.90
social int.	0.06	1.22	0.05	0.59
teamwork	0.01	0.13	0.02	0.24
fairness	0.00	–0.03	0.00	–0.06
leadership	0.08	1.48	0.03	0.40
forgiveness	0.01	0.28	0.14	1.67
humility	0.09	1.63	–0.04	–0.49
prudence	0.07	1.32	0.02	0.21
self-reg.	–0.01	–0.16	0.02	0.20
ABE	0.05	0.94	0.10	1.24
gratitude	0.05	0.87	0.03	0.39
hope	–0.03	–0.54	–0.01	–0.11
humor	–0.02	–0.30	0.01	0.17
spirituality	0.03	0.65	–0.01	–0.15

*Note. N*_Self_ = 365, *N*_VIA Changes_ = 150. Given are effect sizes (based on regression weights, controlled for gender, age, and the duration since the first assessment was completed for VIA Changes). Learning = Love of Learning. Social int. = social intelligence. Self-reg. = self-regulation. ABE = appreciation of beauty and excellence.

*p < .05.

### Research Question 3: Comparing Post-Event Assessments With Differences in Character Strengths

**Figure 4.**
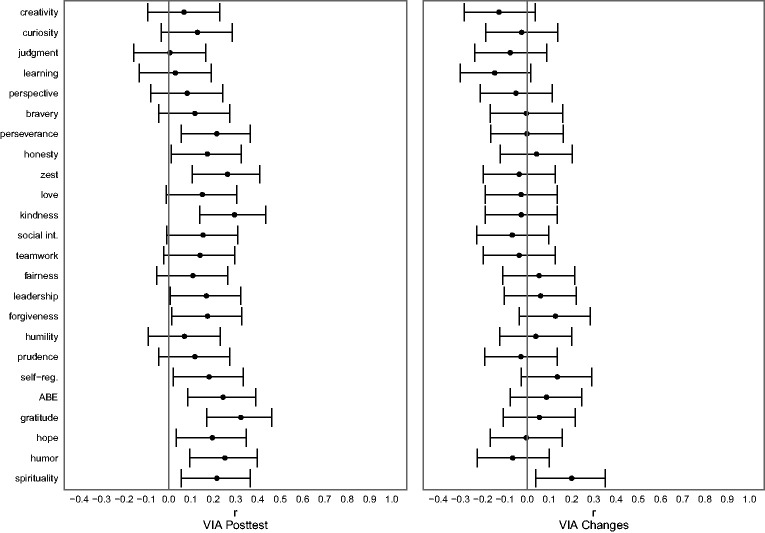
Relationships Between Character Strengths After the First Wave of the Crisis and Changes in Character Strengths With Posttraumatic Growth. *Note. N* = 150. The figures show partial correlation coefficients (controlled for gender, age, and the duration since the first character strengths assessment for VIA changes) with associated 95% confidence intervals. Learning = love of learning. Social int. = social intelligence. Self-reg. = self-regulation. ABE = appreciation of beauty and excellence.

Following the work of Peterson et al. (2008), we analyzed the relationships of character strengths assessed after the event with posttraumatic growth as measured by the PTGI (see Table 3 and Figure 4, left panel). Our results suggested positive associations for 12 strengths: perseverance, honesty, zest, kindness, leadership, forgiveness, self-regulation, appreciation of beauty and excellence, gratitude, hope, humor, and spirituality.

**Table 3.** Research Questions 3, 4, and 5: Relationships Between Character Strengths Before the Crisis (Figure B in Online Repository), After the Crisis, and Changes in Character Strengths With Posttraumatic Growth (Figure 4), and Relationships Between Character Strengths Before Crisis with Compliance and Engagement During the Crisis (Figures 5 and Figure 6).

	Relationships with posttraumatic growth				
	Character strengths before crisis	Character strengths after crisis	Changes in character strengths	Compliance	Engagement
creativity	.07	.07	–.13	–.05	.09
curiosity	.07	.13	–.02	–.04	.12*
judgment	–.05	.01	–.08	.11*	.04
learning	.09	.03	–.15	.07	.04
perspective	.02	.08	–.05	.00	.09
bravery	–.02	.12	.00	.02	.18*
perseverance	.07	.22*	.00	.11*	.06
honesty	.04	.17*	.04	.04	.05
zest	.12*	.26*	–.04	.00	.16*
love	.14*	.15	–.03	–.01	.17*
kindness	.18*	.30*	–.03	.04	.10
social int.	.10	.15	–.07	.02	.12*
teamwork	.09	.14	–.04	.07	.07
fairness	.03	.11	.05	.07	.02
leadership	.05	.17*	.06	.05	.09
forgiveness	.03	.17*	.13	–.03	.06
humility	.04	.07	.04	.14*	–.07
prudence	.04	.12	–.03	.11*	–.10
self-reg.	.00	.18*	.14	.01	.00
ABE	.15*	.24*	.09	.03	–.02
gratitude	.23*	.32*	.06	–.04	.16*
hope	.05	.20*	.00	.03	.13*
humor	.10	.25*	–.07	–.05	.08
spirituality	.18*	.22*	.20*	–.07	.12*

*Note. N* = 365/150/150/365/365. Learning = love of learning. Social int. = social intelligence. Self-reg. = self-regulation. ABE = appreciation of beauty and excellence. Columns 1, 2, 3, and 5 show partial (Pearson) correlations, Column 4 shows partial rank-order correlations. Analyses are controlled for gender and age (Column 2), and additionally duration since the first character strengths assessment (Columns 1, 3, 4, and 5).

*p < .05.

These results were parallel to Peterson et al.’s (2008) reported associations with posttraumatic growth: *r*_s_(22) = .69 (Spearman correlation between the 24 correlation coefficients reported by Peterson et al.’s study and those found in the present study). However, the associations for the correlations between posttraumatic growth and differences in strengths (Figure 4, right panel) were much smaller in size and only reached significance for spirituality. When we compared these relationships with the coefficients reported by Peterson et al. (2008), we found that the overlap between the patterns of relationships was considerably smaller, *r*_s_(22) = .33 (Spearman correlation between the 24 correlation coefficients reported by Peterson et al., 2008, and the associations between posttraumatic growth with changes in character strengths in the present study). Table 3 also shows the relationships of character strengths assessed before the crisis with reported posttraumatic growth.

### Research Question 4: Character Strengths Before the Crisis and Compliance

**Figure 5.**
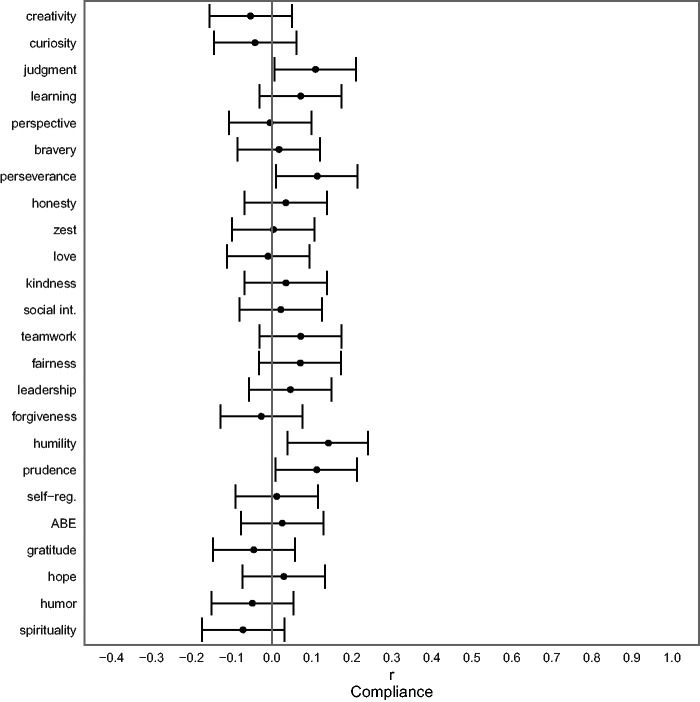
Relationships Between Character Strengths Before the Crisis and Compliance With Regulations During the Crisis. *Note. N* = 366. The figure shows the partial rank-order correlation coefficients (controlled for gender and age) with associated 95% confidence intervals. Learning = love of learning. Social int. = social intelligence. Self-reg. = self-regulation. ABE = appreciation of beauty and excellence.

The relationships of character strengths before the crisis and compliance during the crisis are presented in Figure 5 (see Table 3 for test statistics). Due to the non-normality of the compliance scores, partial Spearman rank-order correlations are given.

Figure 5 demonstrates that participants with higher pre-crisis levels of judgment, perseverance, humility, and prudence reported better compliance with government regulations.

### Research Question 5: Character Strengths Before the Crisis and Engagement in Volunteering

**Figure 6.**
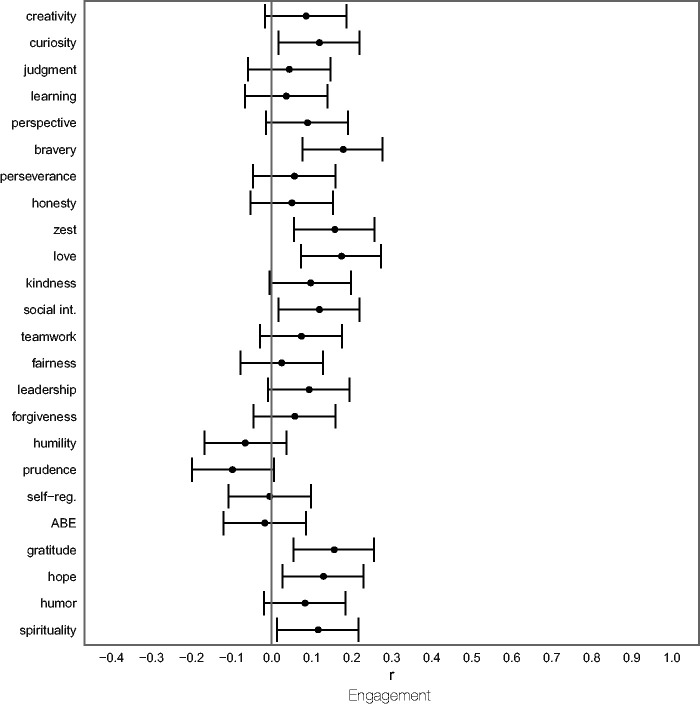
Relationships Between Character Strengths Before the Crisis and Engagement in Volunteering During the Crisis. *Note. N* = 366. The figure shows partial correlation coefficients (controlled for gender and age) with associated 95% confidence intervals. Learning = love of learning. Social int. = social intelligence. Self-reg. = self-regulation. ABE = appreciation of beauty and excellence.

The associations between character strengths before the crisis and engagement in volunteering during the crisis (no/yes) are displayed in Figure 6 (see Table 3 for test statistics). Positive correlations can be interpreted as higher scores among those participants who indicated having volunteered.

The results shown in Figure 6 demonstrate that higher pre-crisis levels of curiosity, bravery, zest, love, social intelligence, gratitude, and hope were associated with volunteering during the crisis.

## Discussion

The present study aimed to investigate character growth and the relevance of character strengths for prosocial behavior as specific instance of growth during the COVID crisis. First and foremost, our study shows that the different data sources considered for character growth (i.e., (1) measured changes in character strengths before and after the first wave of the COVID crisis and (2) retrospectively perceived changes in either oneself or others) did not converge well, which is in line with previous findings (e.g., Frazier et al., 2009). While relying on retrospectively perceived changes leads to the suggestion that almost all character strengths would increase, our measured differences show that only two of the character strengths increased. Since the data sources only showed a minor overlap, we conclude that retrospectively perceived changes in personality traits, both in oneself and in others, are not a useful approach for studying growth.

Prudence and humility were the only two character strengths that showed effects across the different data sources. The increase in prudence seems plausible, since government regulations and recommendations might have served to prime people to pay more attention to their behavior, be more careful, and consider the potential consequences of their actions. Similarly, the recommended behaviors may also have fostered humility, as they required people to put their individual interests aside for the benefit of society at large. This might have led to a stronger tendency to view the self as a part of a larger system, which in turn can foster humility.

Over time, these changes in character strengths might have resulted in trait-level increases (see Fleeson, 2001). We hasten to emphasize, however, that we do not think that changes in self-assessments provide adequate evidence to reach any conclusions regarding sustainable changes in personality traits. The study of personality development requires the use of different methods, such as experimental approaches, or at least, supplementing self-reports with informant reports (see also Jayawickreme & Blackie, 2014). Nonetheless, our results lead us to believe that if any of the character strengths were affected by the COVID crisis, prudence and humility are the most likely ones to have been affected. Thus, we conclude that while people do perceive positive personality changes in themselves and in others following major life events, this does not seem to be supported (or only to a much lesser degree) when the traits themselves are assessed before and after an event (in line with Frazier et al., 2009). We posit, then, that a large part of the perceived changes may be due to retrospective biases.

We also examined whether the individual impact of the COVID crisis moderated changes in character strengths. Overall, the results did not reveal a consistent pattern. If major life events trigger character growth, one would expect to find a relationship between the event’s impact and changes in character (i.e., there should be a “dose-response relationship”). However, this relationship does not necessarily have to be linear; one might also hypothesize that growth happens predominantly in cases with a substantial impact. Being heavily affected by an event might actually prevent an individual from experiencing positive personality changes. While the present study failed to demonstrate any role played by the individual impact of the crisis, this may have been due to limited power for testing interaction effects, given that the main effects of the crisis were relatively small. Moreover, impact is not the only characteristic of (collective) life events that might moderate their effects. Future studies might explicitly assess the perceived characteristics of life events to further understand their role in posttraumatic growth. Luhmann et al. (2020) suggested nine characteristics of life events (valence, impact, predictability, challenge, emotional significance, change in worldviews, social status changes, external control, and extraordinariness), several of which might be relevant in this context.

Furthermore, the present results suggest that character strengths are relevant predictors of specific instances of growth expressed in prosocial behaviors, that is, civic engagement and volunteering as acts of interpersonal help for those in need during a crisis. Levels of curiosity, bravery, zest, love, social intelligence, gratitude, hope, and spirituality before the crisis were higher in those individuals who volunteered during the crisis than they were in those who did not. This finding demonstrates the relevance of studying positive traits as predictors of prosocial behavior that can occur in response to adverse events (“altruism born of suffering”; Vollhardt, 2009).

Our results also suggest that those with higher scores in judgment, perseverance, humility, and prudence tended to show collective helping by following government regulations and recommendations (aiming at protecting oneself and others from infection) more rigorously than those with lower scores. Therefore, these particular character strengths may predict compliance. Although the effect sizes were small by conventional standards, these results extend previous findings (e.g., Aschwanden et al., 2021; Zajenkowski et al., 2020; Zettler et al., 2021) and might bear some relevance for practical purposes. For instance, health campaigns might be addressed specifically to those people who tend to show lower compliance. These campaigns might be developed to share messages tailored to the less humble by emphasizing health benefits for oneself and suggesting positive effects with regard to one’s impression on others when one follows the regulations.

Finally, our study allowed for direct comparison with earlier studies that only examined assessments after an event. Had we only analyzed the relationships of posttraumatic growth with the levels of character strengths assessed after the first wave of the COVID crisis, we would have obtained similar results to those of Peterson et al. (2008), who studied several traumatic events. However, most of these relationships disappeared when we looked at longitudinal changes in character strengths. Thus, we conclude that it is crucial that any research on personality changes following life events or posttraumatic growth considers the longitudinal perspective (see also Jayawickreme & Blackie, 2014; Lamade et al., 2020). While we acknowledge that this renders the study of rare or non-predictable life events very difficult, drawing inferences from cross-sectional data remains highly questionable.

Nonetheless, we think that character strengths offer an important opportunity for studying growth following life events or adversity since there is a strong conceptual connection between character strengths and dimensions of growth as suggested by Tedeschi and Calhoun (1996). The present study also supports the notion that character strengths are personality traits for which people perceive changes following life events and that people believe are affected by crisis. While the PTGI has often been criticized (in line with the present study’s findings and the work of Frazier et al., 2009) for its retrospective assessment, using character strengths in studies on posttraumatic growth might allow assessing similar concepts across multiple occasions (e.g., measuring before and after an event).

Of course, this study had several limitations that must also be addressed. The present study used two measurement time points, one before and one after the first wave of the crisis, which can be considered an important addition to the existing literature. However, using more measurement points—including a time point during the crisis, for example—would have offered more insight into the evolution of potential changes. Also, having two time points does not allow distinguishing separating true change from measurement error (e.g., Singer & Willett, 2003). Since we only had two time points in the present study and therefore analysis options were limited, we used difference scores to examine change because of their simplicity and in line with previous research on the topic (e.g., Frazier et al., 2009). However, difference scores have often has been criticized, for example for their unreliability or their relationship with the pre-test scores (see Gu et al., 2018 for a current overview and a discussion of misconceptions). Thus, for future studies, more time points are recommended which would allow the use of more sophisticated analytical strategies, such as growth models or similar (Singer & Willett, 2003). Furthermore, the present study did not use an experimental approach. While we did test a quasi-experimental approach by examining the potential moderation effects of the individual impact of the crisis, this attempt yielded no conclusive results. Some kind of control, however, is necessary if definite conclusions about the impact of major life events on personality are to be drawn (see also Lamade et al., 2020). In addition, we did not assess individual life events that occurred between the first and second assessments of character strengths as we assumed that life events resulting in increases and decreases in character strengths would be equally likely across the sample. This decision might have added noise to our data.

It should be noted that we used a relatively superficial one-item measure to evaluate compliance with government regulations, which only assessed the extent to which participants had followed their respective government’s recommendations. We used such a broad question because our sample included participants from different German-speaking countries that had experienced varying governmental recommendations. While other studies on compliance with COVID-related regulations used similar one-item measures (e.g., Zajenkowski et al., 2020), it would be informative to assess the compliance with different recommendations separately. Also, we did not control for the severity of the crisis or the severity of government regulations at the participants’ place of residence – both variables might have affected our findings. In addition, our sample was predominately well educated and residing with others, and therefore might have had many resources in these areas that could have buffered the potential traumatic impact of the crisis.

Finally, the present study relied on self-reports and reports on close other persons, which cannot be considered, as already mentioned, proof of true personality change—supplementing self-reports with peer-reports or objective behavior indicators would allow for more convincing conclusions with regard to personality change.

## Conclusion

We conclude that (1) while people may perceive character growth following a major life event, few actual changes are found when self-assessments before and after the event are compared. Retrospective or post-event assessments of oneself or others do not allow reliable conclusions about personality changes following major life events since they only mildly converge with longitudinal changes. We also hold that (2) character strengths were widely unaffected by the COVID crisis with the exception of humility and prudence, which showed increases from before to after the first wave of the COVID crisis. Finally, our study reveals that (3) the levels of several character strengths that had been assessed before the crisis were predictors of instances of interpersonal and collective helping, that is, engagement in volunteering (i.e., curiosity, bravery, zest, love, social intelligence, gratitude, and hope) and compliance with government regulations and recommendations (i.e., judgment, perseverance, humility, and prudence) during the COVID crisis.
